# FTLD-TDP with motor neuron disease, visuospatial impairment and a progressive supranuclear palsy-like syndrome: broadening the clinical phenotype of TDP-43 proteinopathies. A report of three cases

**DOI:** 10.1186/1471-2377-11-50

**Published:** 2011-05-10

**Authors:** Robert Rusina, Gabor G Kovacs, Jindřich Fiala, Jakub Hort, Petr Ridzoň, Iva Holmerová, Thomas Ströbel, Radoslav Matěj

**Affiliations:** 1 Department of Neurology, Thomayer Teaching Hospital and Institute for Postgraduate Education in Medicine, Prague, Czech Republic; 2 Institute of Neurology, Medical University of Vienna, Vienna, Austria; 3 Department of Neurology, Charles University, 2nd Medical Faculty and Motol Teaching Hospital, Prague, Czech Republic; 4 International Clinical Research Center, Brno, Czech Republic; 5 Centre of Gerontology and Faculty of Humanity Studies, Charles University in Prague, Prague, Czech Republic; 6 Department of Pathology and Molecular Medicine, Thomayer Teaching Hospital, Prague, Czech Republic; 7 Department of Neurology and Centre of Clinical Neuroscience, First Faculty of Medicine, Charles University in Prague, Czech Republic

## Abstract

**Background:**

Frontotemporal lobar degeneration with ubiquitin and TDP-43 positive neuronal inclusions represents a novel entity (FTLD-TDP) that may be associated with motor neuron disease (FTLD-MND); involvement of extrapyramidal and other systems has also been reported.

**Case presentation:**

We present three cases with similar clinical symptoms, including Parkinsonism, supranuclear gaze palsy, visuospatial impairment and a behavioral variant of frontotemporal dementia, associated with either clinically possible or definite MND. Neuropathological examination revealed hallmarks of FTLD-TDP with major involvement of subcortical and, in particular, mesencephalic structures. These cases differed in onset and progression of clinical manifestations as well as distribution of histopathological changes in the brain and spinal cord. Two cases were sporadic, whereas the third case had a pathological variation in the progranulin gene 102 delC.

**Conclusions:**

Association of a "progressive supranuclear palsy-like" syndrome with marked visuospatial impairment, motor neuron disease and early behavioral disturbances may represent a clinically distinct phenotype of FTLD-TDP. Our observations further support the concept that TDP-43 proteinopathies represent a spectrum of disorders, where preferential localization of pathogenetic inclusions and neuronal cell loss defines clinical phenotypes ranging from frontotemporal dementia with or without motor neuron disease, to corticobasal syndrome and to a progressive supranuclear palsy-like syndrome.

## Background

Amyotrophic lateral sclerosis (ALS) and frontotemporal dementia (FTD) caused by frontotemporal lobar degeneration with ubiquitin and transactive response DNA binding protein 43 (TDP-43) positive inclusions (FTLD-TDP) represent two different manifestations of the same neurodegenerative disease [1]. Distribution of TDP-43 related neuropathological changes include various anatomical regions with differences in the predominance of lesions, suggesting that TDP-43 proteinopathies reflect the spectrum of a multisystem disorder [2-4]. Mutations in the progranulin (*PGRN*) gene, located on chromosome 17, have been linked to inherited forms associated with neuropathologically detectable TDP-43 proteinopathy and with different clinical symptomatology [5-7]. Moreover, an increasing number of variations within *PGRN *have been described http://www.molgen.ua.ac.be/FTDMutations.

Progressive supranuclear palsy (PSP) is characterized by early gait disturbances and falls, axial rigidity, vertical gaze palsy, and subcortical dementia [8]. PSP is considered to be a tauopathy; however, a PSP-like syndrome has been associated with ubiquitin-only-immunoreactive neuronal changes [9], and thus could be part of the clinical picture of "ALS-plus syndrome" caused by TDP-43 proteinopathy [2,10]. Moreover, FTLD-TDP may have a presentation similar to corticobasal degeneration [11] indicating that these disorders can present with a spectrum of clinical phenotypes that also overlaps other neurodegenerative disorders.

Here we describe three cases, including one with a variation in the *PGRN *gene, with a clinical presentation reminiscent of PSP ("PSP-like syndrome") in patients with FTLD-TDP (with or without MND) that supports the notion of a further, clinically distinguishable, phenotype within the spectrum of TDP-43 proteinopathies.

Clinical and neuropathological data were obtained from three patients followed in the Departments of Neurology, Thomayer Teaching Hospital and Motol Teaching Hospital, Prague, CZ. Patients were assessed using standardized diagnostic tools: magnetic resonance (MRI), electromyography (EMG), routine blood analysis, and neuropsychological assessments. All procedures were explained to the patients and their caregivers, and informed consent was obtained in all cases. All data were analyzed with respect for patient privacy and the protocol was approved by the Ethics Committee of Thomayer Teaching Hospital.

During autopsy, formalin-fixed, paraffin-embedded blocks were obtained from the following regions: frontal, cingulate, temporal, parietal and occipital cortices and subcortical white matter, basal ganglia, thalamus, hippocampus, cerebellum, and brainstem. For immunohistochemistry 5 μm thick sections of formalin-fixed and paraffin-embedded tissue were used with primary antibodies against the following antigens: anti-tau AT8 (1:200, Pierce Biotechnology, Rockford, IL, USA, pS202/pT205); ubiquitin (1:200, rabbit polyclonal; Dako, Glostrup, Denmark); protein p62 (1:4,000, guinea pig polyclonal; Progen Biotechnik GmbH, Heidelberg, Germany); protein TDP-43 (1:2.000, mouse monoclonal; Abnova Corp., Taipei, Taiwan, and 1:100, polyclonal; ProteinTech Group, Chicago, Il, USA), and phospho-TDP-43 (1:2.000, mouse monoclonal, pS409/410; Cosmo Bio Co. Ltd, Tokyo, Japan). Semiquantitatively (0 = none; 1 = mild or few; 2 = moderate; 3 = severe or many), we scored different types of inclusions in selected brain areas.

Genomic DNA was extracted from frozen bone marrow samples (autopsy) using an isolating kit. The microtubule-associated protein (*MAPT*) exon 1-10, transactive response DNA binding protein (*TARDBP*) exons 2-6, progranulin (*PGRN*) exons 0-12 and respective flanking intronic regions were amplified as described previously. Products were examined using agarose gel electrophoresis, treated with ExoSAP-IT (USB, Cleveland, OH, USA), asymmetrically amplified using the DTCS Quick Start Kit (Beckman Coulter, Fullerton, CA, USA), and analyzed on a CEQ 8000 GeXP Genetic Analysis System (Beckman Coulter). The resulting sequences were compared to published *TARDBP, MAPT*, and *PGRN *sequences http://www.ncbi.nlm.nih.gov.

## Case presentation

**Figure 1:**
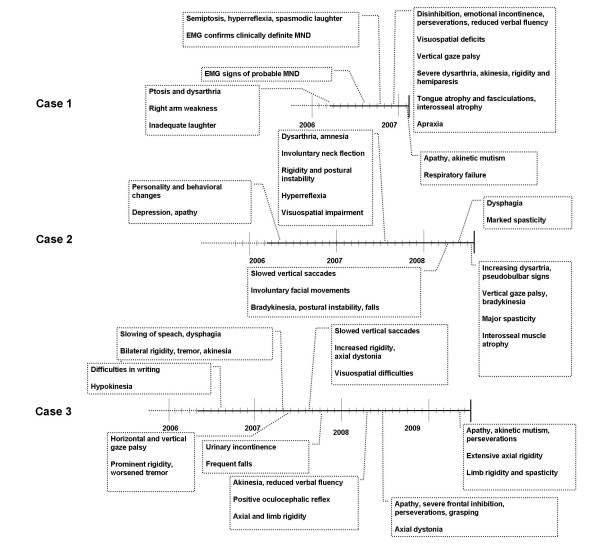
**Temporal evolution of symptoms and features**. Bold lines at the end of the horizontal time line indicate deceases.

Clinical data of the three patients are summarized in (Table 1) and the temporal evolution of symptoms in (Figure 1).

**Table 1:** Summary of clinical presentations

	Case 1	Case 2	Case 3
Age of onset	72	49	64
Duration (months)	18	25	29
Clinical appearance (syndrome)	MND/PSP/FTLD	FTLD	FTLD/PSP

Central motoneuron dysfunction	Hyperreflexia	Generalized spasticity	Right-sided spasticity
Periferal motoneuron dysfunction	Yes, EMG proven	Interosseal amyotrophy	No
Dysarthria/dysphagia	Yes, prominent	Yes, later	Yes-early/prominent
Early falls	Yes	Yes	Yes
Eyelid opening apraxia	Early	Late	Present
Oculomotoricity, conjugate ocular pursuit movements	Abnormal/Voluntar movements reduced both horisontally and vertically	Initially normal, reduced mainly vertically, but after one year	Reduced mainly vertically
Saccades	Slow, hypometric	Slow, hypometric	Slow, hypometric
Bradykinesia	Axial predominance	Axial and limbs	Axial predominance
Rigidity	Axial predominance	Axial and limbs	Axial predominance
Tremor	No	No	No
Dystonia	No	Facial movements, neck anteflexion and lateroflexion	Late - retrocollis
Response to L-dopa	Poor	Poor	Non
Executive dysfunction	Prominent	Prominent	Prominent
Insight	Lacking	Present at the beginning	Lacking
Frontal release signs	Grasping/spastic laughter/disinhibited behavior	Behavioral changes, dysexecutive syndrome	Behavioral changes
Memory problems	Minimal	Minimal, effect of cueing	Impaired
Visuospatial dysfunction	Impaired	Impaired	Impaired
Gait apraxia	Early feature	Present early	Early
Autonomic function	N/A	Not present	Not present
	Case 1	Case 2	Case 3

MRI	Frontotemporal atrophy with left predominance	Generalized atrophy with mild predominance periventriculary and frontal	Frontotemporal and brainstem atrophy
Cerebrospinal fluid	Normal	Not done	Normal
EMG	Probable ALS according to El Escorial criteria, normal conduction	Not done	Normal conduction, no denervation

Pathological diagnosis	FTLD-TDP/MND	FTLD-TDP/MND	FTLD-TDP/MND

### Case 1

A 70-year-old woman was referred to our department for spontaneous ptosis and slowly (five months) progressive dysarthria. In the family, apart from her two children with multiple sclerosis, there was no history of neurologic disease.

Clinical examination revealed asymmetric semiptosis, paralytic dysarthria, and mild finger (right hand) weakness. Reflexes were brisk and there was no sensory impairment. Needle EMG showed chronic regeneration and fibrillation patterns in the anterior tibialis and common finger extensor muscles. Nerve conduction studies and repetitive stimulation were normal.

Gradually, disinhibition, emotional incontinence, and spasmodic laughter developed. Five months later a neurological examination revealed blepharospasm instead of ptosis. Frontal lobe involvement (reduced verbal fluency, stereotypias, perseverative features, grasping, and apathy) became associated with impairment in visuo-spatial functions and short-term memory. Akinesia with rigidity, but without tremor, was more pronounced in axial muscles; reflexes remained brisk. There was noticeable slowing of oculomotor saccades, but without gaze palsy.

**Figure 2:**
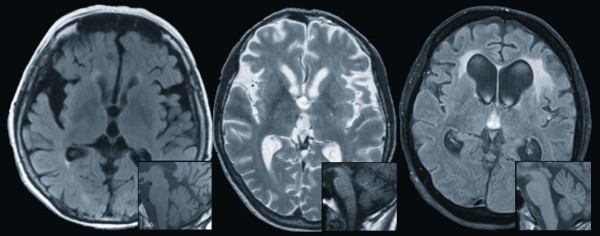
**Magnetic resonance images**. MRI show an asymmetric frontotemporal atrophy on transversal scans in case 1 (left, FLAIR sequence), case 2 (middle, T_2 _sequence), and case 3 (right, FLAIR sequence). In inboxes a relatively preserved brain stem is demonstrated on sagittal sections.

Over the next two months, the patient's condition progressively deteriorated. Extrapyramidal features and dysexecutive signs worsened, accompanied by frequent falls. The blepharospasm diminished and vertical gaze palsy developed together with eye-lid closing apraxia. Dysarthric features progressed and frontal-type gait apraxia with astasia-abasia, and incontinence appeared. A brain MRI showed marked asymmetric frontotemporal atrophy (Figure 2).

Neuropsychological assessment revealed relatively preserved episodic verbal memory and language, with conserved learning abilities (Token Test and Boston Naming Test). Executive functions were markedly impaired (Wisconsin Card Sorting Test: one category, more than 16 perseverative errors; Trail Making Test: under 10^th ^percentile for age-matched norms). The patient failed in immediate and delayed recall of the Rey-Osterrieth Complex Figure; basic visuospatial skills and copying were altered to a lesser degree.

Repeated EMGs, showing signs of widespread denervation and chronic reinervation, confirmed clinically definite ALS, according to the El Escorial criteria [12]. Treatment with riluzole was started, but with little effect on disease evolution. Because of increased dysphagia and malnutrition, a percutaneous endoscopic gastrostomy (PEG) was performed six weeks later. The patient deceased two months later from terminal bronchopneumonia.

### Case 2

A 49-year-old woman, without comorbidities, was referred for cognitive and behavioral changes. Two years prior, her husband had noticed personality changes accompanied by difficulties in complex task solving (cooking, grooming). Her mood was slightly depressive and apathetic; polydipsia had also been noticed by the family (6 liters a day), but no metabolic or endocrine abnormalities were found. There was a history of dementia in the family involving a grandmother, who died at the age of 92.

Six months later we observed gait instability, swallowing difficulties and involuntary neck anteflexion. Asymmetric, left-predominant, rigidity displaying the cogwheel phenomenon and postural instability were present. Reflexes were brisk. There was no gaze palsy or fasciculations. MRI scans showed generalized atrophy with only mild, nonspecific white matter changes (Figure 2).

The neuropsychological profile was primarily dysexecutive (Frontal Assessment Battery, Digit Span, Verbal Fluency Test, and Trail Making Test under 10^th ^percentile for age-matched norms). Language analysis showed mild alteration (Boston Naming Test 6 mistakes), visuospatial functions were impaired (Clock Drawing Test - incorrect placement and repeating of numbers, Rey-Osterrieth Complex Figure with respect to copy but severely altered recall). We also noted important episodic memory impairment with reduced learning abilities sensible to cuing: The Auditory Verbal Learning Test (AVLT): 16 words - 5 retrieved spontaneously, 11 more with cuing) and occasional confabulations. The Mini Mental State Examination (MMSE) score was 22/30.

Vertical gaze palsy appeared 10 months later, with severe Parkinsonism and frequent falls. Levodopa (3 × 62.5 mg/day) had no effect and was stopped after 1 month because of progressive dysphagia. The MMSE dropped to 16/30 and the Activities of Daily Living (ADL) scale revealed complete loss of functional capacity. Two months later, pseudobulbar signs with significant dysphagia and weight loss (15 kg in 6 months) and increasing spasticity with hyperreflexia and pyramidal signs were reported. The patient received memantine with no effect on MMSE or ADL, citalopram was added to treat signs of mild depression.

Two months later (after nearly two years of evolution), her condition deteriorated rapidly to profound dementia and severe Parkinsonism. Interosseal muscle atrophy was found, but no fasciculations; reflexes remained brisk. An EMG was scheduled but never completed. Shortly after admission there was a sudden decline in her condition, she died of bronchopneumonia and septic shock.

### Case 3

A 64-year-old woman, with a family history of stroke (mother), developed gait instability, dysarthria, and mnestic difficulties over a 2 year period. Muscle rigidity appeared, falls occurred, and she became incontinent. Six months later, in another hospital, multiple system atrophy was diagnosed, and treatment with levodopa and pramipexole was started. Escitalopram was added for depression a few months later. Despite increasing doses of levodopa (up to 1000 mg daily), rigidity and akinesia worsened and the patient became wheelchair-bound. Ultimately, she was referred for diagnostic re-evaluation.

Clinical examination found psychomotor slowing, dysexecutive features with apathy, perseverations, and stereotypical behavior. Verbal fluency was decreased and there was a marked lack of insight. Speech required effort and was associated with palilalia and fluctuating dysphagia. There was no gaze paralysis, but horizontal and vertical saccades were slowed; fixation was impaired and eyelid-opening apraxia was observed. Extrapyramidal features included extensive axial rigidity with significant akinesia, but no tremor. Notable right-sided hypertonus was associated with extremity spasticity.

A MRI revealed significant atrophy with frontal and temporal predominance, and periventricular dilatation, and brain stem atrophy (Figure 2). The EMG was difficult to evaluate (extensive rigidity and lack of cooperation); there was suspicion of denervation potentials.

A cognitive assessment was incomplete due to lack of cooperation. However, we found signs of noticeably impaired executive functions and behavioral changes, visuo-constructional abilities and episodic memory were impaired with relatively conserved cueing effect (figure copying, cued recall).

Despite high-doses of L-dopa (1.5 g daily) and baclofen (150 mg daily) the condition worsened over the next months. Subcortical dementia with severely impaired fluency progressed to anarthria, and finally mutism. Massive perseveration interfered with any spontaneous movement and spasticity increased to flexion contractures. Oculomotor involvement included gaze apraxia, dissociation between impaired voluntary gaze and preserved involuntary horizontal pursuit, together with a freely elicitable oculocephalic reflex. Rigidity and akinesia remained notable in axial regions, later accompanied by dystonia and retrocollis. The patient's condition continued to decline and she deceased shortly thereafter from urosepsis.

Genetic analysis revealed a pathogenic mutation (102 delC (predicted protein: Gly35GlufsX19)) in the *PGRN *gene previously described [3]. We found no variations in *TARDBP *and *MAPT *genes.

### Neuropathology

**Figure 3:**
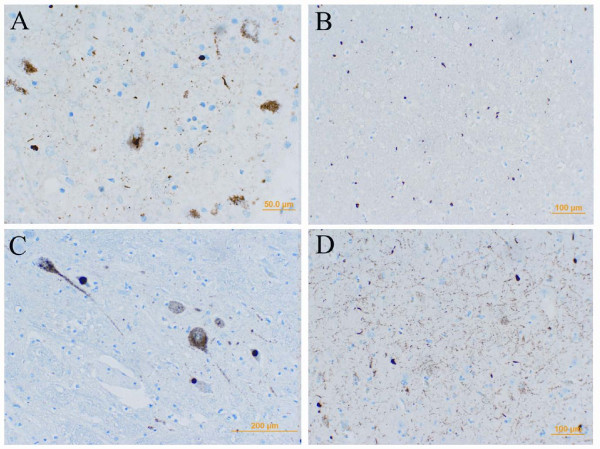
**Immunohistochemistry for phospho-TDP-43**. Spherical, "Lewy-like" and skein-like neuronal cytoplasmic inclusions as well as diffuse granular inclusions were found in substantia nigra (case III - A), pontine base (case I - B) and in hypoglossal nucleus (case II - C); thin and long as well as globular neurites in hippocampal formation (case I - D). Scale bars = Bar graphs: 50 μm (A), 100 μm (B and D), 200 μm (C).

Neuronal loss and gliosis were most prominent in the mesencephalon, basal ganglia, and frontal and temporal cortices. TDP-43 and phospho-TDP-43 immunostaining revealed neuronal cytoplasmic inclusions of different types, including compact globular and dot-like structures, more pathological profiles were detected using anti-phospho-TDP-43 antibody. Neuritic profiles, positive for ubiquitin and p62 of variable intensity, were seen in different brain structures (Figure 3). Glial cytoplasmic inclusions and thread-like (phospho)-TDP-43 immunoreactivity was seen in the white matter. These cases did not strictly fit the subtyping schemes recently proposed [13]. However, due to the slight predominance of neuronal cytoplasmic inclusions in the frontal cortex and hippocampus Case 1 and 2 were reminiscent, following the proposed classification, of type 2. The long and tortuous neurites as well as neuronal cytoplasmic inclusions, seen in Case 3, made it more compatible with type 3 according to Cairns et al. [14]. Neuronal nuclear inclusions, associated with type 4, were not convincingly observed in any of cases. In addition skein-like neuronal inclusions were seen in brainstem nuclei in all cases, including substantia nigra (all), hypoglossal nucleus (Cases 1 and 2) and inferior olivary nucleus (Case 3).

**Figure 4:**
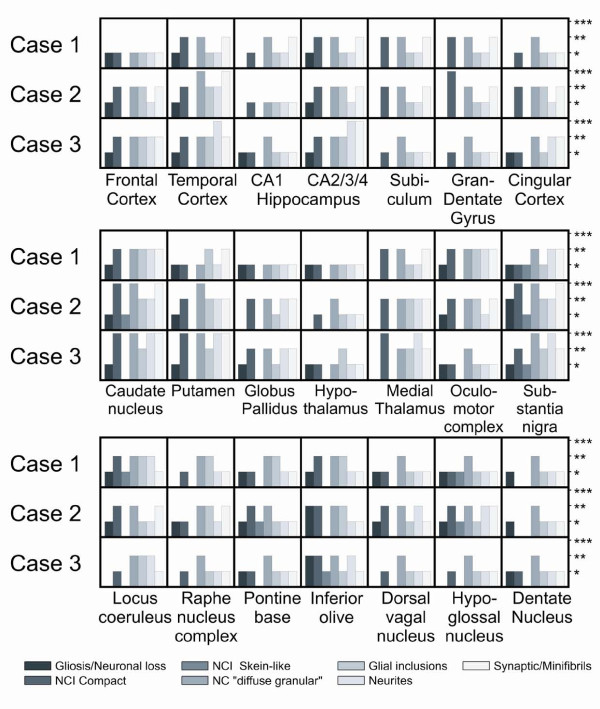
**Distribution of pathological changes within the brain tissue**. Evaluation: (*) mild, (**) moderate and (***) high presence of a given pathological feature. NC - neuronal cells, NCI - neuronal cell inclusions, Gran-Dentate Gyrus - dentate gyrus, granular layer

In summary, we found hallmarks of FTLD-MND with ubiquitin-immunoreactive neuronal changes and intracytoplasmic and neuritic TDP-43, phosphorylated TDP-43, and p62 immunopositivity (Figure 3) compatible with the diagnosis of FTLD-TDP with MND. Detailed mapping of immunohistochemical positivity in different brain areas is summarized in (Figure 4).

## Conclusions

We present three autopsy proven cases of FTLD-TDP with clinical manifestation as "progressive supranuclear palsy plus" syndrome. A summary of our study observations include: (*i*) during the clinical course, but not as initial symptoms, all patients showed clinical hallmarks of PSP (subcortical dementia, axial predominant akinesia and rigidity, supranuclear gaze abnormalities, and frequent falls), (*ii*) additionally, upper and/or lower motor neuron involvement and a frontal behavioral syndrome ("ALS-plus" syndrome) were also detected, (*iii*) FTLD-TDP was confirmed neuropathologically in all cases, (*iv*) mutations in *MAPT, TARDBP *were not observed, while in one patient we found a *PGRN *mutation (Case 3), and (*v*) the extent of histopathological changes correlated positively with progression and were widespread in subcortical structures.

All cases shared some common features, although there were also some important differences (Table 1, Figure 1 and 2).

Case 1 was classified as clinically definite ALS within the first year. Frontal lobe involvement with behavioral changes and a "PSP-like" syndrome with Parkinsonism and supranuclear ophthalmoplegia developed only later. Illness duration was 14 months. Neuronal inclusions were common in the hippocampal and temporal areas, caudate nucleus, thalamus, and oculomotor complex, and in the inferior olive, while TDP-43 immunoreactive neurites and glial inclusions and neuronal loss was prominent in the caudate nucleus, locus coeruleus, substantia nigra, and oculomotor nuclei. Skein-like inclusions were detected in the hypoglossal nucleus.

In contrast, the "PSP-like" syndrome in Case 2 appeared earlier, was associated with behavioral signs of FTD and Parkinsonism, progressed slowly to dementia and severe rigidity with supranuclear gaze palsy. The clinical course was longer (4 years) and lower motor neuron signs came very late. Neuronal inclusions (including skein-like neurites and glial pathology) were more widespread and strongly associated with subcortical and brainstem nuclei.

Case 3 had a clinical picture that was most typical for PSP, linked to FTD and upper motor neuron involvement. Neuronal inclusions and neurites and glial inclusions had dispositions similar to Case 2 (hippocampal areas, fronto-temporal cortex, caudate, putamen, substantia nigra). A pathogenic mutation (102 delC) in the *PGRN *gene was also detected.

Clinical manifestations, age of onset, duration of illness and distribution of neuropathological changes of *PGRN *gene mutation carriers are variable: the majority have progressive aphasia or frontotemporal dementia, but without symptoms of MND or PSP [13,15]. Familial forms of FTLD expressing prominent parkinsonian features have been linked to mutations in *MAPT*, formerly frontotemporal dementia and Parkinsonism linked to chromosome 17 (FTDP-17) [16]. We did not observe tau pathology and *MAPT *gene analysis excluded mutations in all presented cases. More recently, *MAPT *negative cases with a familial setting were shown to harbor mutations in the *PGRN *gene [17]. These cases were neuropathologically identified as tau negative but ubiquitin-positive [5,18]. The clinical and neuropathological phenotype and age of disease onset, tends to be highly variable not only among families but even between individuals in one family [19]. Case 3 expands this group to include the PSP-like phenotype, in addition to primary progressive aphasia [20] or corticobasal syndrome [21].

In summary, criteria for probable PSP [8] were fulfilled in Cases 1 and 2 (Table 1). In Case 3, all signs were present, but the evolution lasted more than one year. Overall, features of PSP developed later in the course of our patients. It must be noted that none were diagnosed clinically as PSP. The severe frontal and temporal atrophy on MRI (Figure 2) was oriented more towards FTLD; however all displayed considerable features of PSP. It is noteworthy, that in addition to subcortical dementia, typical for PSP [22], all of our patients developed considerable behavioral and personality changes compatible with behavioral variants of FTD. This becomes most apparent in Case 3 (apathy, perseverations, stereotypical behavior, lack of insight and dynamic aphasia), however, rigidity, oculomotor findings, considerably restricted verbal fluency and the absence of disinhibition or compulsivity are less typical of FTD. MRI scans of our patients showed only mild asymmetry of atrophy and the degree of frontal and temporal atrophy was not as impressive as in many other FTLD-TDP-43 cases. The subcortical white matter changes in Case 3 are similar to findings in *PGRN *mutation carriers [23]. Finally, MRIs in our patients did not show posterior fossa findings evoking PSP (such as hummingbird sing, thinning of superior cerebellar peduncle or prominent mesencephalic atrophy).

All patients corresponded to possible/probable MND with significant upper motor neuron signs in Case 3, lower motor neuron involvement in Case 2, and both in Case 1 [12]. The extent of histopathological changes (Figure 4) correlated positively with progression.

The distribution of histopathological changes in particular TDP-43 immunoreactive profiles involved subcortical and brainstem structures somewhat more than reported previously, in association with FTLD-TDP subtypes [24], supporting the notion that TDP-43 proteinopathies are representatives of a multisystem proteinopathy [25] with involvement of anatomical regions corresponding to predominant clinical symptoms. The progression rate of clinical features differed in our cases (Figure 1).

An important finding in our patients is the impairment of visuospatial functions in all three cases. This profile is rather uncommon in parkinsonian syndromes. A large prospective cohort of patients with PSP and multiple system atrophy (MSA) demonstrated the existence of a cognitive profile similar to that previously reported in idiopathic Parkinson´s disease. Visuospatial functions scores, measured on the dementia rating scale (DRS), for construction and conceptualization, were largely preserved [26]. Another study suggested that visuospatial functions are not consistently impaired in atypical parkinsonian syndromes, but the degree and pattern varied across the diseases. This could imply a different neural basis in each condition, since the most prominent visuospatial impairment was linked to corticobasal degeneration [27]. There are other findings however, which suggest that visuospatial and visuoperceptual dysfunctions reflect structural grey matter changes in temporo-parietal cortical regions of Parkinson´s disease patients [28].

Severe visuospatial impairment is not a characteristic of motor neuron disease. Patients with FTLD-MND and ubiquitin inclusions whose visuospatial skills were tested by copying drawings and the visual object and space perception battery (VOSP) subtests, did not show remarkable alterations [29].

On the other hand, visuospatial deficits have been reported in FTLD patients. Koedam et al. recently published a very interesting observation; neuropsychological profiles, including visuospatial impairment, does not differ between frontotemporal dementia patients with and without lobar atrophy, although patients without atrophy, on MRI, were less severely demented [30]. Other authors have suggested that parietal deficits including visuospatial dysfunction may be a prominent feature of *PGRN *mutations [31].

A very similar observation to ours demonstrated a movement disorder resembling PSP and associated with dementia and MND-like pathology. Besides selective verbal and action processing, there was visual recall and recognition (Door and People test (DPT)) and lower construction and conceptualization scores (VOSP). The most important neuropathological findings were abundant ubiquitin-positive inclusions, which were also detected in areas without apparent atrophy or neuronal loss [32].

There have been only very limited reports of oculomotor abnormalities in FTLD-MND. In our cases we found significant neuropathological changes in the mesencephalic tegmentum. This supports the notion that vulnerability patterns may differ in TDP-43 proteinopathies (sporadic and genetic forms) leading to variable clinical phenotypes. Indeed, our observations with several previous studies indicate that sporadic TDP-43 proteinopathies may present as MND, FTD, progressive aphasia, FTD with MND, corticobasal syndrome [11], and PSP-like phenotypes associated with FTD and features of MND. These clinical phenotypes may show overlap with tauopathies; indeed PSP (tauopathy) may be also associated with MND and corticospinal tract degeneration [33]. Furthermore, these clinical phenotypes were reported in cases with an established genetic cause, linked not only to *PGRN *mutations but also to mutations in *TARDBP*. MND, FTLD-MND, and FTD with supranuclear palsy having also been associated with *TARDBP *variations [34-36], demonstrating the complexity of clinico-pathological and genetic relations.

In conclusion, our observation suggests that: (*i*) association of "PSP-like" to "ALS-plus" syndromes in the same patient may represent a clinically distinguishable entity within a broad spectrum of TDP-43 positive neurodegeneration; (*ii*) this clinical manifestation develops irrespective of genetic variation in *TARDBP, MAPT*, and *PGRN*; (*iii*) the clinical picture seems to be related to preferential localization of pathogenetic inclusions and neuronal cell loss rather than specific pathological mechanisms of the disease itself; and (*iv*) motor neuron involvement, visuospatial and frontal behavioral signs should be systematically explored in atypical PSP patients.

## Consent

Written informed consent was obtained in one case from the patient and in two cases from patient´s relatives for publication of this case report and any accompanying images. A copy of the written consent is available for review by the Editor-in-Chief of this journal.

## Abbreviations

(FTD): Frontotemporal dementia; (FTLD-TDP): Frontotemporal lobar degeneration with ubiquitin and TDP-43 positive neuronal inclusions; (FTLD-MND): Frontotemporal lobar degeneration associated with motor neuron disease; (ALS): Amyotrophic lateral sclerosis; (TDP-43): Transactive response DNA binding protein 43; (*TARDBP*): Transactive response DNA binding protein; (*PGRN*): Progranulin; (*MAPT*): Microtubule-associated protein; (PSP): Progressive supranuclear palsy; (MRI): Magnetic Resonance Imaging; (EMG): Electromyography; (AVLT): Auditory Verbal Learning Test; (MMSE): Mini Mental State Examination; (ADL): Activities of Daily Living; (VOSP): Visual Object and Space Perception battery; (MSA): Multiple System Atrophy; (DRS): Dementia Rating Scale

## Competing interests

The authors declare that they have no competing interests.

## Authors' contributions

RR, JF and RM made substantial conceptual contributions to the design of the study, analysis of sources, and contributed to drafting of the manuscript. GGK was involved in data analysis and gave critical revision of the manuscript regarding important intellectual content. RM and GGK performed neuropathological and immunohistological examination. TS performed and discussed the results of genetic analysis. PR, IH and JH supplied important and relevant clinical data of the patients. All authors have read and approved the final version of the manuscript.

## Pre-publication history

The pre-publication history for this paper can be accessed here:

http://www.biomedcentral.com/1471-2377/11/50/prepub
